# The Expanding Role of Systemic Therapy in the Management of Hepatocellular Carcinoma

**DOI:** 10.1155/2018/4763832

**Published:** 2018-08-07

**Authors:** Omar Abdel-Rahman, Winson Y. Cheung

**Affiliations:** ^1^Clinical Oncology Department, Faculty of Medicine, Ain Shams University, Cairo, Egypt; ^2^Department of Oncology, University of Calgary, Tom Baker Cancer Centre, Calgary, Alberta, Canada

## Abstract

Hepatocellular carcinoma (HCC) represents a global health problem, with the majority of patients presenting at an advanced or incurable stage. The development of effective systemic therapy options for this disease has been challenging because many HCC patients suffer from underlying liver cirrhosis that precludes the safe delivery of systemic therapy. The current review seeks to provide an overview of the current systemic therapeutic approaches for advanced HCC as well as some of the novel management strategies that are currently being evaluated.

## 1. Introduction

Primary liver cancer represents a global health problem since it is the sixth most common cause of cancer as well as the second most common cause of cancer mortality worldwide [1]. Hepatocellular carcinoma (HCC) represents more than 90% of cases of primary liver cancer [2].

The development of cirrhosis precedes tumor formation in most cases and as expected most causes of cirrhosis represent the main risk factors for developing HCC. These include mainly hepatitis B and C, chronic alcoholism, aflatoxin exposure, and other rarer causes of cirrhosis such as hemochromatosis [3]. The association between all etiologic forms of cirrhosis and HCC is illustrative of geographic imbalance in the incidence of HCC worldwide. For example, hepatitis B is endemic to parts of Asia [3].

According to international guidelines focusing on the management of HCC, the diagnosis of HCC should be based on well-defined imaging criteria and/or histological confirmation. A number of screening programs have been implemented worldwide in order to detect cases of HCC at an earlier stage anticipating that this strategy can lead to improvements in patient outcomes [4].

Effective HCC management relies on multidisciplinary involvement in the decision-making process and takes into account the various patient-related, disease-related, and treatment-related factors [5]. This is reflected in the various staging systems for clinical decision-making in HCC where in contrast to the majority of solid tumors, the traditional TNM staging system is not commonly utilized [6]. The staging system validated and used most frequently for outcome prediction and treatment allocation is the Barcelona Clinic Liver Cancer (BCLC) staging system [7]. For early-stage cases among fit patients, potentially curative treatments (e.g., resection, transplantation, and ablation) represent the standard of care [8]. For intermediate-stage cases, transarterial chemoembolization and other locoregional therapies might be used [9, 10]. Conversely, for advanced-stage disease (portal vein invasion or extrahepatic disease) in patients with preserved liver function, systemic therapy options represent the primary treatment modality.

Unfortunately, most cases of HCC are detected at an advanced or incurable stage. In the context of currently available treatment modalities, overall survival for many of these cases does not exceed one year [11]. Improvement in the outcomes of advanced-stage disease is thus the focus of most current scientific efforts.

The current review aims to provide an overview of the available standard of care systemic treatments for HCC, as well as offering some insights into new investigational approaches.

## 2. Current First-Line Therapy of Advanced-Stage Disease (Stage C BCLC)

Systemic therapy is the current standard of care for advanced-stage disease. Sorafenib (a multikinase inhibitor) was the first agent to demonstrate overall survival and received regulatory approval in 2007. Its approval was based principally on two landmark international phase III studies, the SHARP study and the Asia-Pacific study [12, 13]. Both studies randomized patients with advanced HCC into either treatment with sorafenib or placebo. Both studies limited their inclusion to patients with good performance status, adequate liver function, and minimal comorbidity. In the SHARP study, there was a statistically significant improvement in overall survival with sorafenib compared to placebo (median overall survival was 10.7 months in the sorafenib group and 7.9 months in the placebo group; HR, 0.69; 95% CI, 0.55 to 0.87; P=0.00058). Similarly, in the Asia-Pacific study, there was a statistically significant, albeit smaller, benefit in overall survival with sorafenib compared to placebo (median overall survival was 6.5 months in patients treated with sorafenib, compared with 4.2 months in the placebo group; HR 0.68; 95% CI 0.50-0.93; P=0.014).

Subsequent secondary analyses of phase III studies have shown that lower ALBI (albumin/bilirubin) score, ECOG (Eastern Cooperative Oncology Group) performance score of 0, BMI (Body Mass Index) ≥ 25, AFP (Alpha-fetoprotein) < 200 ng/ml, and no extrahepatic spread are associated with better overall survival among patients treated with sorafenib [14]. Interestingly, sorafenib appears to be of greater benefit for HCC patients with underlying hepatitis C virus infection compared to hepatitis B virus infection [15].

Subsequently, several other studies have been conducted in order for other targeted agents (e.g., brivanib and sunitinib) to be studied in comparison to sorafenib. However, it was demonstrated that the studied agents were nonsuperior or equal to sorafenib and additionally had a less favorable side effects profile and poorer tolerability [16, 17]. Other studies have also evaluated different sorafenib-based combinations (e.g., sorafenib plus erlotinib, sorafenib plus doxorubicin); however, there was no superiority in terms of survival [18, 19].

An important caveat for the interpretation of the above is the fact that the vast majority of patients included into the two landmark studies had well-preserved liver function (Child-Pugh class A) and good performance status. This is very important as the majority of HCC patients encountered in routine clinical practice are typically significantly frailer. They usually have advanced cirrhosis, poor liver function, and a compromised functional status. Accordingly, extrapolation of these data from clinical trials into the real world should be done cautiously. More recently, lenvatinib (multikinase inhibitor) was compared in a phase III study to sorafenib in the first-line treatment of unresectable HCC. This study showed that lenvatinib was noninferior to sorafenib with regard to overall survival [20].

## 3. Current Second-Line Therapy of Advanced HCC (after Failure of Sorafenib)

Several other cytotoxic and targeted agents have also been tested as second-line treatment. However, most of these studies have not proven to be successful due to lack of efficacy and/or poor tolerability [21–23]. In 2016 regorafenib, another multikinase inhibitor became approved in this setting. The approval of regorafenib was based on a landmark phase III study which compared regorafenib versus placebo in patients who tolerated and progressed on sorafenib. Regorafenib improved overall survival (HR 0·63; 95% CI 0·50-0·79; P<0·0001); median overall survival was 10·6 months for the regorafenib group versus 7·8 months for the placebo group [24]. However, it should be noted that patients included in the above study were also highly selected (i.e., good performance status and Child A liver function), which is in contrast to the vast majority of advanced HCC, post-sorafenib patients encountered in routine clinical practice. Furthermore, regorafenib has significant toxicities which may preclude its use in most real-world patients. Specific side effects include hand-foot syndrome, fatigue, and hypertension. Overall, the use of regorafenib in this fragile patient population with borderline liver function needs to be cautiously approached.

More recently, the results of another landmark trial (CELESTIAL study) comparing cabozantinib versus placebo as second-line treatment were published. Cabozantinib is a multikinase inhibitor with potent inhibitory activity against c-MET [25]. In the CELESTIAL phase III study, cabozantinib improved overall survival compared to placebo. Median overall survival was 10.2 months for cabozantinib versus 8.0 months for placebo (HR 0.76, 95% CI 0.63-0.92; P=0.0049) [26]. It is expected that cabozantinib will be soon adopted as an alternative second-line treatment for advanced HCC following progression on sorafenib. The favorable outcomes in the placebo-treated group likely underscore the fact that trial patients were probably highly selected. Thus, caution should again be exercised when generalizing these results into routine clinical practice.

A third agent which has recently drawn much attention in the second-line treatment of advanced HCC is nivolumab which is a PD-1 inhibitor. The results of a phase 1/2, open-label, noncomparative, dose escalation, and expansion trial (CheckMate 040) were recently published [27]. In this study, previous sorafenib treatment was allowed. The objective response rate was 20% (95% CI 15–26%) in patients treated with nivolumab 3 mg/kg in the dose-expansion phase and 15% (95% CI 6–28%) in the dose-escalation phase. As a consequence, the FDA has granted nivolumab conditional approval as a second-line agent. The results of a phase III study (CHECKMATE 459) comparing nivolumab versus sorafenib in treatment-naïve HCC are anticipated, alongside a number of other ongoing studies.

Following the favorable results of nivolumab, a number of other immune checkpoint inhibitors are currently being evaluated. The interest in immune checkpoint inhibitors was stimulated by several preclinical findings suggesting that HCC is an immunogenic tumor with numerous tumor-associated antigens [28]. Moreover, there has been work focusing on elucidating the HCC tumor microenvironment with early suggestions of the presence of an upregulated PD(L)1 pathway in HCC [29]. This has led to a plethora of ongoing clinical trials investigating PD(L)1 inhibitors (alone or in combination with other treatments) in the management of HCC. As of April 2018 and on the clinicaltirals.gov registry, there are 23 open clinical trials investigating nivolumab and 19 investigating pembrolizumab in the management of HCC.

Before adopting the use of immune checkpoint inhibitors in the management of these patients, it is noteworthy that these agents are also known to produce a wide spectrum of immune-related adverse events including cutaneous, hepatic, gastrointestinal, endocrine, and pulmonary adverse events [30–34]. Given the impaired liver function seen in the majority of patients with HCC, appropriate and thorough assessment of the tolerability and pattern of adverse events among these patients (particularly with respect to hepatic and gastrointestinal adverse events) needs to be conducted. Previous reports have suggested that the use of immune checkpoint inhibitors may potentially lead to viral hepatitis reactivation [35]. The extent to which immune checkpoint inhibitors can be safely administered in HCC patients is not yet known with certainty.

## 4. Investigational Role of Systemic Therapy in Earlier Stages of HCC

### 4.1. Adjuvant Systemic Therapy

The use of adjuvant systemic therapy following resection, transplantation, or locoregional ablation drew attention among oncologists and hepatologists treating patients with HCC. However, all relevant studies to date have failed to provide a convincing benefit to justify their routine use in clinical practice [36].

### 4.2. Systemic Therapy in Combination with Locoregional Transarterial Treatments

Several studies have also evaluated various combinations of systemic therapy (particularly sorafenib) with locoregional treatments (including transarterial (chemo)-embolization and radio-embolization). The stated rationale for such combinations is the potential of transarterial treatments to induce a consequent surge in VEGF levels following the procedure, and this was presumed to be the main pathogenetic process behind treatment failure [37]. Thus, this resulted in significant interest towards the concurrent administration of VEGF-VEGFR targeted agents, which was hypothesized to decrease the rates of failure by countering this mechanism. Despite some interesting preliminary findings, the overall evidence provided is not yet convincing enough to justify the widespread use of this approach [38, 39].

## 5. Conclusions and Future Directions

Outcomes of patients with advanced HCC are still very poor. The development of novel therapeutic approaches to improve overall survival and quality of life is an unmet need. The introduction of sequential targeted therapeutics including sorafenib as first-line treatment and regorafenib, cabozantinib, and nivolumab as second-line treatment could contribute to improved outcomes for these patients. Nevertheless, severe side effects and poor tolerability pose a real challenge and the vast majority of advanced HCC patients in clinical practice have compromised performance status and/or poor liver function. To date, prevention of liver cirrhosis remains the most effective way to prevent development of HCC. For instance, there has been significant reduction of hepatitis B-related HCC following the introduction of hepatitis B vaccination. Future research should be directed towards a better understanding of the biology of the disease and of the complexity of the biological process within its microenvironment. Agents targeting a single pathway or a single pathogenetic process within HCC have not been very successful in combating this disease. We are in need for more unique and strategic approaches which may include various combinations of treatments or modalities.
